# Retrieving the size distribution of SBA-15 mesopores from small-angle X-ray scattering data using a Monte Carlo method

**DOI:** 10.1107/S160057672300691X

**Published:** 2023-09-01

**Authors:** Xiangyin Tan, Barbara Bianca Gerbelli, Márcia Carvalho de Abreu Fantini, Cristiano Luis Pinto Oliveira, Heloísa Nunes Bordallo, Pedro Leonidas Oseliero Filho

**Affiliations:** a Niels Bohr Institute, Copenhagen University, Denmark; b Centro de Ciências Naturais e Humanas, Universidade Federal do ABC, São Paulo, SP, Brazil; c Instituto de Física, Universidade de São Paulo, São Paulo, SP, Brazil; d European Spallation Source, Lund, Sweden; Argonne National Laboratory, USA

**Keywords:** Monte Carlo methods, SAXS, small-angle X-ray scattering, data modelling, SBA-15

## Abstract

A Monte Carlo (MC) method was introduced into a state-of-the-art model used to analyse small-angle X-ray scattering (SAXS) data of SBA-15, an ordered mesoporous material with many applications. With this new procedure, referred to herein as the SBA-15+MC model, it is possible to retrieve the size distribution of the mesopores, *D*(*r*), in a free modelling approach. To achieve this, two main points were addressed: (i) based on previous implementations, the method was adapted to work with long core–shell cylinders; (ii) since the MC model requires longer processing times, strategies to speed up the calculations were developed, which included a simplified version of the original model used to analyse SAXS data of SBA-15 (referred to as the SBA-15 model) as well as the determination of several structural features from the SAXS curve prior to the fit. The new model was validated with simulated data and later used to fit experimental SAXS curves of SBA-15. The obtained results show that the SBA-15 model only works well because the mesopore size distribution of SBA-15 is narrow, whereas the new approach can be successfully used in cases where *D*(*r*) is wider and/or has a more complex profile, such as SBA-15 with expanded mesopores. Even though a specific SAXS example was chosen to prove the model, the strategies presented herein are general and suitable for inclusion in other models aimed at the analysis of SBA-15 and similar ordered mesoporous materials.

## Abstract 2

The new model proposed in this work allows retrieval of the mesopore size distribution of SBA-15 (and similar ordered mesoporous materials) from small-angle X-ray scattering data using a free modelling approach. According to the obtained results, even more complex size distributions can be recovered, such as the case of SBA-15 with expanded mesopores.

## 1. Introduction

The ordered mesoporous material (OMM) known as SBA-15 has highly ordered pores in the size range 2–50 nm (called mesopores) (Zhao *et al.*, 1998 ▸). Among many applications, it is used as a carrier of drugs (Alazzawi *et al.*, 2021 ▸), enzymes (Losito *et al.*, 2021*a* ▸) and, more recently, vaccines (Fantini *et al.*, 2022 ▸; Oliveira *et al.*, 2022 ▸), in which the desired material is loaded into the mesopores. Because the loading success is mostly size dependent (Kang *et al.*, 2007 ▸; Diao *et al.*, 2010 ▸), it is crucial to characterize in advance the size, shape, size distribution and spatial ordering of the mesopores. Among the experimental techniques used to this end, small-angle X-ray scattering (SAXS) is probably the most suitable and convenient, since the experiments are non-invasive, easy to perform, fast and reproducible (Oliveira, 2011 ▸). Nevertheless, advanced data modelling is needed to retrieve ‘hidden’ structural information contained in the experimental curves, like the size distribution of mesopores. The existing model used to follow the formation of a 2D-hexagonal hybrid material, proposed originally by Sundblom *et al.* (2009 ▸), based on the work of Förster *et al.* (2005 ▸) and later generalized by Manet *et al.* (2011 ▸), gathers all essential physical information and can satisfactorily fit SAXS data of SBA-15 (Losito *et al.*, 2021*a* ▸). However, as in other approaches, this model assumes an analytical expression for the size distribution 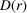 of the mesopore core radius and/or shell thickness (*e.g.* Gaussian, lognormal, Schulz–Zimm *etc*.) which, although realistic, constitutes a strong constraint and makes it impossible to describe, *e.g.*, simple bimodal-like distributions. To accomplish this, one would have to have prior knowledge of the distribution type, for instance using gas adsorption measurements (Thommes & Cychosz, 2014 ▸), and then adapt the equations with this information. The model adaptation, although feasible, would be limited to that specific application.

Aiming for a more general solution to this problem (Tan, 2022 ▸), here we propose a flexible version of the model by using the Monte Carlo (MC) method, already applied to analyse polydisperse spheres and cylindrical nanoparticles (Pauw *et al.*, 2017 ▸). The final aim is to recover the mesopore size distribution in a free modelling approach. However, in contrast to the mentioned application of the MC method, here we must deal with core–shell particles and many additional parameters describing other structural features, such as the lattice parameter, the Debye–Waller factor, which considers distortions of the lattice relative to an ideal one, and peak shape parameters, among others. Moreover, the MC method usually requires longer processing times, which limits its applicability. All combined, this corresponds to a challenging problem and the solutions described here are suitable for SAXS data modelling of SBA-15 and similar OMMs.

## 2. Description of the models

### 2.1. The model (‘full SBA-15 model’)

**Figure 1:**
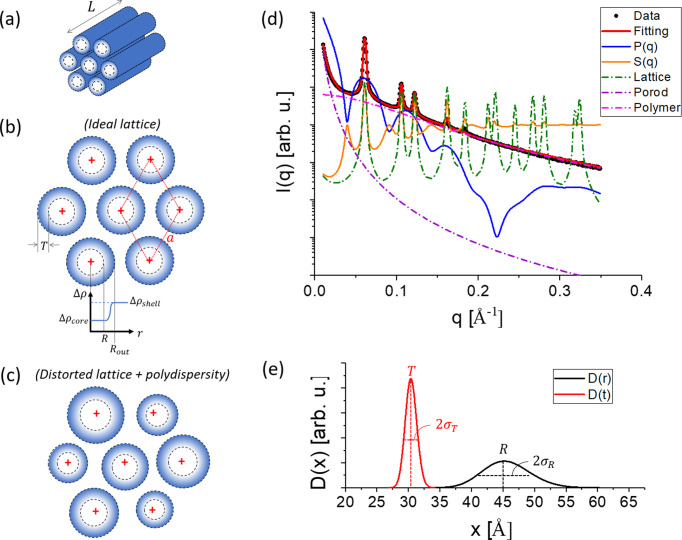
(*a*) Modelling of SBA-15, where the mesopores are represented by cylinders with length *L* arranged in a 2D-hexagonal lattice. (*b*) Front view of modelled SBA-15 with lattice parameter *a* considering an ideal lattice, represented by the red crosses, in which the cylinder centres coincide with the lattice points. The mesopores have average inner and outer radii *R* and 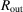 , respectively, with 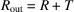 , where *T* is the average thickness of the shell. Core and shell have electron-density contrasts 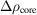 and 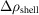 , respectively. (*c*) In a more realistic scenario, besides polydispersity of *R* and 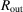 , the lattice can be distorted, *i.e.* the centre of each cylinder does not coincide with the lattice points (red crosses). (*d*) Black filled circles correspond to the SAXS experimental curve of SBA-15 obtained at the CoSAXS beamline (MAX IV, Lund, Sweden), where it is possible to observe Bragg reflections whose indexing is compatible with a 2D-hexagonal lattice (Losito *et al.*, 2021*a* ▸). The red continuous line is the fit with equation (1), which is quite satisfactory. The other continuous lines represent the form factor 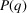 (blue) and the structure factor 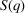 (orange). The green, purple and magenta dashed–dotted lines show the function 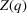 [equation (10)], the Porod term and the polymer scattering [equation (21)], respectively. (*e*) The normalized volume-weighted size distribution of the core radius, 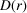 , and the shell thickness, 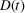 , obtained from the fit, with averages and standard deviations (*R*, 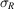 ) and (*T*, 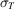 ), respectively.

In the model proposed by Sundblom *et al.* (2009 ▸), SBA-15 is represented as an anisotropic system [Fig. 1 ▸(*a*)] in which long core–shell cylinders of length *L* along the *z* axis have polydisperse cross sections, forming the mesopores, which are embedded in a ⊥-plane forming a 2D-hexagonal lattice characterized by the lattice parameter *a* [Figs. 1 ▸(*b*) and 1 ▸(*c*)]. In this scenario, the scattered intensity 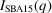 is

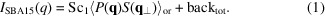

The decoupling approximation (Kotlarchyk & Chen, 1983 ▸), applied to the first term of equation (1), allows the separation of the form factor 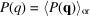 , describing the cylinders’ scattering, and the structure factor 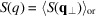 , associated with the lattice, where the brackets 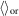 indicate the orientational average over all possible directions in the 3D space and the parameter 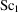 is a scale factor. The term 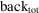 corresponds to a total background and will be discussed later. Assuming that the cylinders are long, the form factor can be approximated as the product of the longitudinal factor, 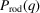 , and the polydisperse core–shell cross-section contribution, 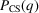 :

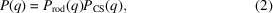

with

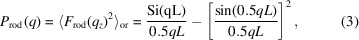




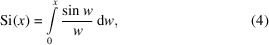

where 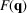 is the amplitude form factor. Since the cylinder’s cross section is circular, the corresponding amplitude form factor 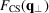 is not dependent on rotation. Thus 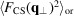 is just the square of 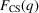 (Glatter & Kratky, 1982 ▸), with

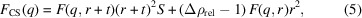




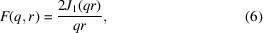




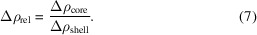


*R* is the core radius and *T* is the shell thickness, 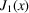 is the first-order Bessel function of first kind, and 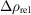 is the ratio between the core and shell electron-density contrasts. The factor 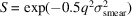 appearing in equation (5) is used to smear the outer interface of the cylinders and to simulate the entrances in the silica matrix left by the polymer template after its removal through calcination (Schwanke *et al.*, 2018 ▸). Polydispersity, represented herein by the notation 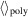 , is included by calculating one extra integral, yielding the final expression of 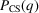 :

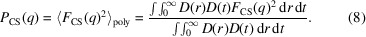




The polydispersity in *R* and *T* is analytically introduced using any distribution function 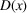 (*x* represents the variable radius *r* and shell thickness *t*), such as Gaussian, lognormal, Schulz–Zimm *etc.* [Fig. 1 ▸(*e*)]. If 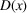 is normalized, the denominator of equation (8) is equal to unity.

The structure factor 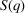 describes the peaks in the SBA-15 SAXS pattern [Fig. 1 ▸(*d*)]:

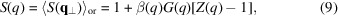

with

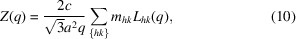




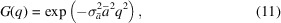




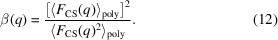

[It was brought to our attention that a more accurate analysis of scattering data for 2D materials might be possible by rigorously and properly deriving equation (9) in the context of the basic assumptions established in the first paragraph of Section 2.1. This is, however, outside the scope of the present article, but may be a good exercise for experts interested in this field.]

**Figure 2:**
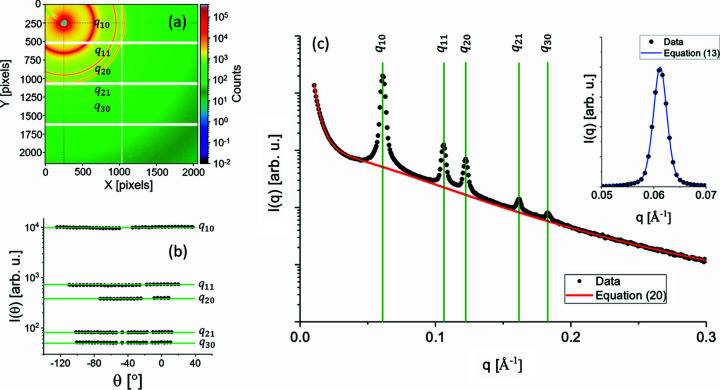
(*a*) 2D scattering pattern of SBA-15 collected at the CoSAXS beamline (MAX IV, Lund, Sweden). (*b*) Azimuthal scan in the region of each ring shown in (*a*), confirming the scattering is isotropic in the probed angular range. Otherwise, large deviations of the intensity 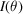 versus the azimuthal angle 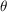 relative to the green lines (a guide for the eyes) would be observed. (*c*) From data reduction, the 1D curve is obtained (black filled circles). It was fitted with equation (20) to determine the total background (continuous red line) and, consequently, the parameters 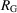 , Sc2, back and AP (see their meaning in Table 1 ▸). The position of each peak (vertical green lines) is satisfactorily predicted by assuming a 2D-hexagonal space group [equation (18)]. Once the total background is evaluated, its subtraction from the experimental data is performed and the first peak is isolated and fitted with equation (13) to obtain the parameters 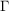 and 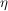 controlling the shape of all peaks in the curve.

The factor 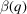 is the influence of the form factor polydispersity on the structure factor, whereas 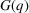 describes lattice distortions caused by positional disorder of the cylinders, the so-called Debye–Waller factor, which is quantified by the mean-square displacement 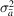 . The parameter 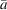 is the next-nearest-neighbour distance between adjacent particles, which corresponds to the lattice parameter *a* for a 2D-hexagonal lattice. The function 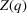 , equation (10), represents the peaks in the lattice, each one with multiplicity 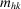 (equals 6 for *h*0 and *h* = *k* reflections or 12 otherwise), where *hkl* are the Miller indices (*l* = 0 for a 2D-hexagonal lattice). The first five diffraction peaks typically observed have Miller indices (10), (11), (20), (21) and (30) with multiplicity 6, 6, 6, 12 and 6, respectively [Fig. 2 ▸(*c*)]. The function 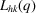 describes the peak shape. Although Förster *et al.* (2005 ▸) proposed the use of a versatile function that shifts from Gaussian to Lorentzian depending on the choice of the ν parameter controlling the peak shape, we observed that a pseudo-Voigt function, corresponding to a simple linear combination between Gaussian and Lorentzian, is faster and more stable. This comes from the fact that it does not require the use of any special function or complex quantity (Losito *et al.*, 2021*a* ▸):

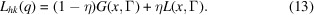




The parameter η varies between 0 and 1 and changes the peak shape from Gaussian to Lorentzian, respectively. 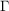 is the full width at half-maximum (FWHM) of the peak and is a common parameter between 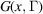 and 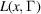 , defined by

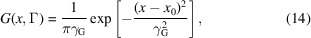




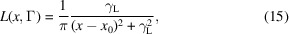

with

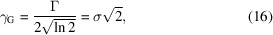




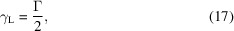

where σ is the standard deviation of the Gaussian and 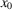 is the peak position. For a 2D-hexagonal system, the position of the peaks 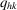 is

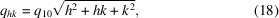

where

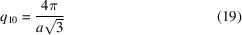

corresponds to the position of the first peak. The parameter *c* in equation (10) is a constant, ensuring that the product of form factor and structure factor fulfils the equation for the Porod invariant (Förster *et al.*, 2005 ▸). Since it is well defined only for sharp interfaces, which is not the case for SBA-15 and other OMMs, it is convenient to use the approach proposed by Manet *et al.* (2011 ▸) and replace *c* by the constant 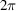 , the result of a crystallographic analysis of the Bragg reflections (Manet *et al.*, 2011 ▸). The remaining term of equation (1) constitutes a total background [Fig. 2 ▸(*c*)],

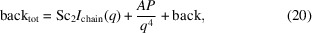

and contains information on the incoherent scattering (back), on the Porod law ∼*q* ^−4^ behaviour at low *q* and on the micropore contribution, 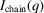 , modelled as a Gaussian chain and due to the polymeric template (Sundblom *et al.*, 2009 ▸):

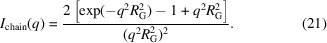




The use of a Porod term was proposed by Manet *et al.* (2011 ▸) with the justification that it comes from the interfaces of the silica grains surrounded by a medium which, in our case, is a vacuum. We observe that this term has great importance for the total background of SBA-15 data, as also demonstrated by a previous study (Pollock *et al.*, 2011 ▸). As a final step, instrumental resolution, represented by the resolution function 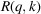 , can be taken into account (Pedersen, 1997 ▸):

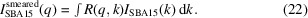




### 2.2. Simplified SBA-15 model (‘SBA-15 model’)

To ensure the feasibility of the MC approach with the model detailed in the previous section, our first goal was to accelerate the calculations, which was achieved by applying a simplified version of the original model, hereafter referred to as the ‘SBA-15 model’. As proposed by Losito *et al.* (2021*a* ▸), we started the generalization of the model by assuming that the experimental data were collected using an X-ray beam with point collimation. For this specific setup, common in all synchrotrons and in most laboratory SAXS instruments, the instrumental smearing, essentially related to the peak broadening, contains information on the domain sizes, on the order of hundreds of nanometres in the case of SBA-15 and other mesoporous materials; it is therefore out of the scale length probed by SAXS and might be disregarded. Without smearing calculations, the processing of the model is much faster. Nevertheless, further optimization was required. Thus, we removed the polydispersity in *T* since it has a small influence on the fit quality, which greatly simplifies the calculation of equations (8) and (12). From this point forward, the size distribution of the mesopores is only related to 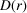 .

In advance of the fit, one can additionally and independently:

(i) Estimate the total background using equation (20) [Fig. 2 ▸(*c*), red continuous line], resulting in the evaluation of 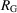 , Sc2, back and AP parameters (see their meaning in Table 1 ▸). To do this, one could fit the region of the SAXS curve without the peaks [in the example shown in Fig. 2 ▸(*c*), we fitted the curve disregarding the points in the range from 0.045 to 0.2 Å^−1^].

(ii) Use the position of the first peak 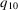 to index the remaining peaks and check if the mesopore arrangement is 2D hexagonal [Fig. 2 ▸(*c*), green vertical lines], thus allowing calculation of the lattice parameter *a* using equation (19).

(iii) Subtract the estimated background from the curve and fit the first peak with equation (13) [inset of Fig. 2 ▸(*c*), blue continuous line] to evaluate the peak shape, described by the parameters Γ and η.

To finalize, on the basis of previous information from the system as well as the general procedure used for small-angle scattering (SAS) analysis, it is advisable to constrain the remaining fit parameters *T*, 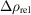 , 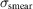 , 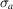 and Sc1 (see Table 1 ▸ for their description). After this careful and optional pre-evaluation, fewer fit parameters remained, saving about 30–50% of the processing time according to our tests.

A summary of all parameters of the SBA-15 model is given in Table 1 ▸. In the next sections we will show, step by step, how the MC model can work with long cylindrical core–shell mesopores.

### 2.3. MC model of long cylinders (‘cylinder+MC model’)

Considering a set of simple cylinders (*i.e.* they are not core–shell), ‘diluted’ [*i.e.* no interparticle interaction, so 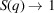 ], with volume 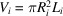 and contrast scattering length 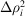 , the scattered intensity is

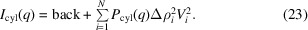




If the length 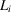 is longer than the probed scale length, it is safe to assume a constant value for all cylinders, for instance, *L* = 1000 Å used in our calculations. On the other hand, it is also reasonable to assume that 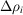 is the same for all cylinders in the set: 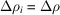 . Using equation (2) for long cylinders, equation (23) is written as

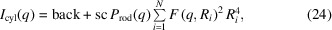

where 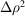 and the factor 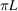 (from 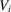 ) were included in the scale factor sc, which allows us to work in a relative scale of intensity. This calculation could be performed in an absolute scale, but it would require *a priori* information on the system, for instance the contrast scattering length of the scatterers (Pauw *et al.*, 2013 ▸).

For convenience, we define the auxiliary function 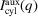 as

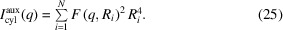

Thus equation (24) is written as

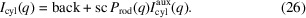




### 2.4. MC model of long core–shell cylinders (‘cylinder-CS+MC model’)

For long core–shell cylinders with a smeared outer interface, the auxiliary function and the corresponding theoretical scattered intensity are

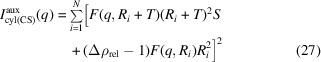




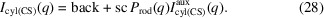

Since the parameters *T* and 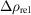 are unknown, solving equation (27) is not as trivial as solving equation (25). Thus, by defining

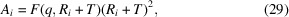




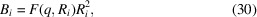

we can rewrite equation (27) as

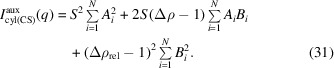

This allows us to determine the array 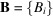 and consequently 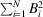 as soon as 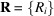 = 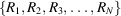 containing all 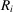 values is created (see Section 2.6), speeding up the calculations. This is not true for 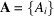 , which depends on *T* being determined along with sc, back and 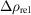 during the optimization procedure.

### 2.5. SBA-15 model + MC (‘SBA-15+MC model’)

Aiming to use an MC method with the SBA-15 model we rewrote equation (1) and equation (12) as

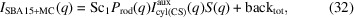




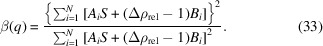




To take advantage of the strategy presented in the previous section, we can write equation (33) in a more convenient way:

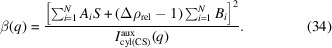

Since the array 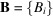 is fully determined as soon as 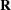 is created, the computation of 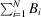 is immediate. As discussed before, the function 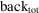 , equation (20), and many parameters of 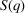 could be estimated in advance and used during the optimization with MC, which saves a considerable amount of time.

### 2.6. Processing MC models

**Figure 3:**
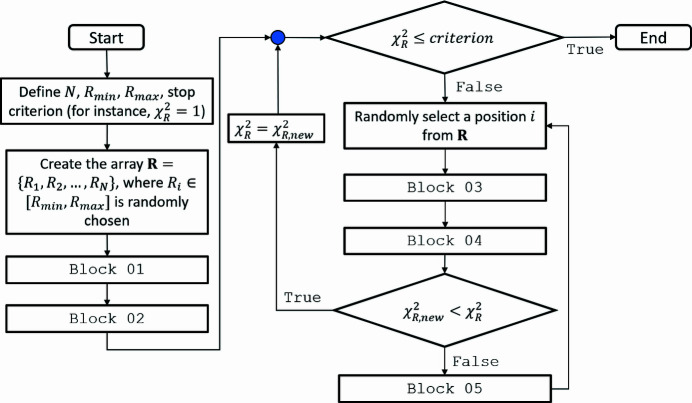
Generalized flowchart of the program for parameter optimization based on the logics presented by Bressler *et al.* (2015 ▸). Each code block is filled with the pertinent code related to the processing of cylinder+MC, cylinder-CS+MC and SBA-15+MC models (Table 2 ▸).

The processing of all presented MC models is straightforward and follows the flowchart depicted in Fig. 3 ▸. Briefly, we start by defining the number of particles *N* used in the simulation as well as the minimum and maximum radius values, 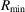 and 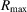 , respectively. At this stage, it is also fundamental to define the stop criterion which, in our case, is the reduced chi-square, 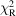 (Pedersen, 1997 ▸):

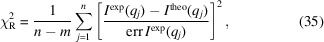

where 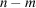 is the number of degrees of freedom, *n* is the number of experimental points and *m* is the number of fit parameters. An ideal fit has 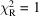 . Thus, we can set this as a general stop criterion. The parameters *N*, 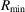 and 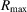 are used to create the array 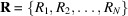 , with the random selection of *N* elements 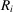 in the interval 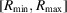 . In the following, two model-dependent code blocks are executed (Table 2 ▸) and will return, using a least-squares residual minimization procedure, the initial value for 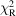 as well as those for the fit parameters in the model. The cylinder+MC model has to determine, for instance, the parameters sc and back, while the SBA-15+MC model has to determine Sc1, *T*, 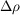 , 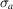 and 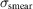 . If 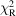 reaches the stop criterion value, then we have already found the solution, *i.e.* the radius distribution 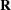 and the fit parameter values. Otherwise, the algorithm will continue its execution by randomly selecting one of the elements of 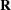 , identified by its position 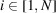 , and changing it by another value randomly selected in the same interval 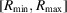 . Again, two model-dependent code blocks are executed (Table 2 ▸) and will return the new values for the fit parameters and 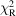 . If the new 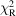 is smaller than the previous one, then the 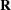 change is accepted. Otherwise, it is directly rejected (Pauw *et al.*, 2013 ▸). The procedure is repeated until the stop criterion is reached.

## 3. Materials and methods

### 3.1. Simulated data

The theoretical scattering is only known for some objects, like spheres and cylinders (Pedersen, 1997 ▸), which is not the case for SBA-15. To check if the SBA-15+MC model is working, two simulated data sets with errors (Sedlak *et al.*, 2017 ▸) were created using equation (1), one with typical values of SBA-15 as input (Garcia *et al.*, 2016 ▸; Losito *et al.*, 2021*a* ▸) and another where the mesopores are expanded (all input values are shown in Table 3 ▸). In both cases, the mesopore size distribution is described by normalized lognormal functions, as done by Losito *et al.* (2021*a* ▸). Regarding the expanded mesopores, we used the work of Guo *et al.* (2019 ▸) as inspiration for the input parameter values (see Table 3 ▸) and for the mesopore size distribution, generated by summing two lognormal distributions with average radius and standard deviation given by (55.0 Å, 7.5 Å) and (78.0 Å, 11.5 Å). In this way, the obtained theoretical size distribution resembles the one experimentally evaluated by means of the nitro­gen adsorption isotherm (NAI) technique (Guo *et al.*, 2019 ▸).

### 3.2. Experimental data

SBA-15 was synthesized as described previously by Losito *et al.* (2021*a* ▸). SAXS measurements were performed at 25°C at the CoSAXS beamline (MAX IV, Lund, Sweden). The scattering patterns [Fig. 2 ▸(*a*)] were collected on a 2D detector (Eiger2 4M, Dectris) and are isotropic in the probed *q* range [Fig. 2 ▸(*b*)]. Data processing (background subtraction, azimuthal averaging) was performed using the dedicated beamline software. The intensities are described as a function of the reciprocal-space momentum transfer modulus *q*, defined as 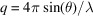 , where 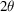 is the scattering angle and λ = 1.00 Å is the radiation wavelength. The sample-to-detector distance was 3032 mm, providing a useful *q* range of 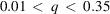 Å^−1^. It is noteworthy that SBA-15 has the potential to become a calibrant material for ultra-small-angle X-ray scattering as the structure is well defined, resulting in sharp, isotropic and concentric rings, as observed in the 2D scattering patterns recorded at the CoSAXS beamline. Illumination of the same region for several seconds showed no statistical variation of the scattering, suggesting that the tetrahedral structure of silica is less prone to structural modification under X-ray irradiation. The robustness of the SBA-15 structure means it can potentially be used as a calibration standard in high-intensity X-ray sources, such as fourth-generation synchrotrons. Further investigation on this aspect is ongoing at the CoSAXS beamline.

### 3.3. MC processing setup

For the fits with the SBA-15+MC model, both simulated and experimental curves have 200 points, equally and linearly distributed in the range 0.01 < *q* < 0.30 Å^−1^, which allows distances between 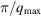 ∼ 10 Å and 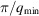 ∼ 300 Å to be probed. For MC fits, we chose *R*_min_ = 20 Å and *R*_max_ = 80 Å, which covers the SBA-15 mesopore typical size, about 50 Å radius, except when analysing simulated data of SBA-15 with expanded mesopores, where we used *R*_max_ = 140 Å. The number of particles was set to 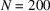 particles while the size distribution histogram is obtained from the binning of the final 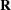 array using 40 bins, the same setting as used by Pauw *et al.* (2013 ▸) based on the sampling theorem (Pauw *et al.*, 2013 ▸). Of course, this can be arbitrarily changed to lower or higher values. Because MC is a stochastic process, we quantified the statistical variation by means of error bars in each bin indicating the sample standard deviation over ten repetitions. This statistical analysis was performed not only for the histograms but also for the fit parameters determined through least squares in the optimization. Thus, by running the SBA-15+MC model several times, one can evaluate the average and standard deviation for each bin of the histogram as well as for each fit parameter.

The MC code was written in Python and the calculations were performed on an Intel Core i5-10400 desktop computer running at a clock speed of 2.9 GHz. With this setup, the models cylinder+MC, cylinder-CS+MC and SBA-15+MC take approximately 1 s, 1 min and 30 min per iteration, respectively, to be executed. It might be possible to speed up the processing even further if one uses, for instance, pre-compiled code. Another strategy could be the use of fewer points to describe the *q* range (for instance, 100 instead 200 as in this work). Nevertheless, one should be careful not to lose important details in the regions of the curve corresponding to the peaks, which have in general fewer points compared with the other parts of the curve. In this context, the use of a subroutine to smartly filter out and/or interpolate points would be preferable.

## 4. Results and discussion

**Figure 4:**
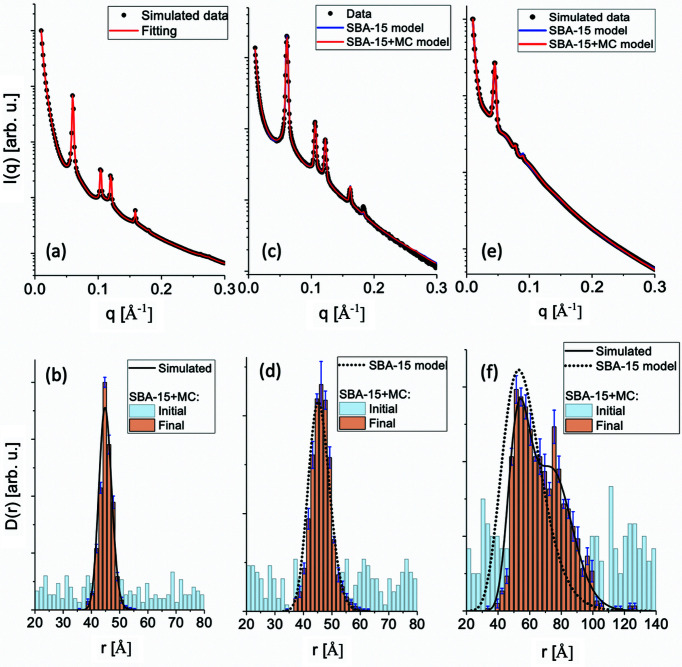
(*a*) Simulated SAXS data of SBA-15 (black filled circles) using typical values as input (Table 3 ▸), fitted with the SBA-15+MC model (red continuous line). (*b*) Mesopore size distribution obtained using the SBA-15+MC model procedure superimposed onto the 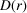 function used as input for the simulated data (black continuous line). The fit with the MC procedure was repeated ten times, which allowed us to estimate the statistical variance of each histogram bin. For comparison purposes, we show the histogram of one of the initial uniform distributions used in the optimization (light-blue bars). (*c*) Experimental SAXS data of SBA-15 (black filled circles) fitted with both the SBA-15+MC (red continuous line) and SBA-15 models (blue continuous line). (*d*) Mesopore size distribution [as described in the caption for (*b*)] obtained using the SBA-15+MC model superimposed onto the function 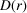 determined by the SBA-15 model. (*e*) Simulated SAXS data of SBA-15 with expanded mesopores (black filled circles) using the values given in Table 3 ▸ as input. The fit was performed with both the SBA-15+MC (red continuous line) and SBA-15 models (blue continuous line). (*f*) Simulated size distribution (black continuous line) superimposed onto mesopore size distribution [as described in the caption for (*b*)] obtained using the SBA-15+MC model superimposed onto the function 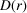 evaluated by the SBA-15 model.

In Fig. 4 ▸(*a*) is shown the fit of the simulated data with the SBA-15+MC model. The fact that the fit is almost perfect ( 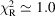 ) is to some degree expected because, as discussed before, fundamentally the same model is used to produce and to fit data. Despite that, the result shows the correctness of the calculations and, most importantly, the ability of the SBA-15+MC model to successfully retrieve the mesopore size distribution without any *a priori* information [Fig. 4 ▸(*b*)]. Moreover, the model is capable of returning values of the fit parameters *T*, 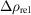 , 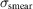 , Sc1 and AP which are in satisfactory agreement with the ones used to generate the simulated data (Table 3 ▸).

In the context of this validation, we observed in a few cases the non-convergence of the method when using larger 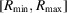 intervals. Considering this, our recommendation is that one constrains not only the fit parameters but also the 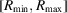 interval as much as possible to ensure both convergence and meaningful results. By doing this, one can also slightly speed up the calculations, since the sampling space for MC processing is reduced.

After the validation of the SBA-15+MC model, we proceeded with the fit of experimental data of SBA-15. The result is shown in Fig. 4 ▸(*c*). For comparison purposes, we also fitted the data with the SBA-15 model. As we can observe, the two fits are practically overlapped, having close 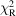 values and, within error bars, similar values for the fit parameters (Table 3 ▸), which are in agreement with results reported in the literature (Garcia *et al.*, 2016 ▸; Losito *et al.*, 2021*a* ▸). The value of 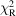 is not unity in either case, because it was not reachable during the fits. In the optimization of the SBA-15+MC model, the cycle was stopped as soon as the parameter 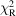 started to change very slowly. Interestingly 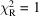 , besides being a general stop criterion, is not always fulfilled when fitting experimental data, even when the data uncertainties are well estimated. This happens simply because any model, corresponding to a simplification of the investigated system, might not be able to completely describe all structural features of the system in the probed length scale. For instance, if the system is composed of non-interacting spherical nanoparticles with uniform scattering length contrast, the model of polydisperse spheres captures all structural features and, in this case, it is possible to fulfil 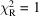 (Yang *et al.*, 2020 ▸). The same model used to study slightly more complex systems, such as the ones formed by polymeric spherical micelles, now cannot describe all features of the system and deviations of the fit compared with experimental data, quantified by 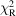 , start to occur. This is likely in our case, where the SBA-15 model, although very detailed, is not able to describe all features of the system. As one can observe in Fig. 4 ▸(*c*), the region of curve from *q* ∼ 0.17 Å is not well fitted, particularly the fifth peak, in agreement with some previous studies that used this model (Garcia *et al.*, 2016 ▸; Losito *et al.*, 2021*a* ▸,*b* ▸). This points to the need for further improvement of the model itself, which is out of the scope of this work. Thus, it is clear that 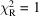 , corresponding to the minimum stop criterion, is not always reachable, raising the question of what could be a good value for the stop criterion. Besides the fact that one has total freedom to define it, especially when the data uncertainties are over- or underestimated (Bressler *et al.*, 2015 ▸), from the MC optimization it is quite easy to observe when 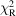 reaches an asymptotic behaviour and starts to decrease very slowly. In this context, a tolerance for termination by the change of 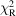 can be set. For instance, the optimization process is stopped when 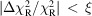 , where 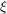 , as 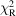 , is arbitrarily defined and 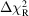 is the difference between the current and the previous value of 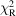 . In our case, we used 10^−8^ as the default, and in all tests the fit was satisfactory, with both size distribution and fit parameters presenting reasonable values.

The retrieved size distribution using the SBA-15+MC model is in good agreement with the one obtained with the SBA-15 model [Fig. 4 ▸(*d*)]. Therefore, the two approaches yielded similar results. For this specific application, since the SBA-15+MC model is more general, it validates the choice of the analytical size distribution used in the SBA-15 model [dotted lines in Fig. 4 ▸(*d*)], which is likely only reasonable because SBA-15 has a very narrow and defined size distribution, as indicated by gas adsorption measurements (Losito *et al.*, 2021*a* ▸,*b* ▸). This is not necessarily true for SBA-15 with expanded pores (Garcia *et al.*, 2016 ▸; Dacquin *et al.*, 2012 ▸; Guo *et al.*, 2019 ▸), for instance, which justifies the model flexibility proposed in this work.

To address this point, simulated data of SBA-15 with expanded pores were generated using experimental assays detailed by Guo *et al.* (2019 ▸) as inspiration. The input parameters are shown in Table 3 ▸, and the simulated size distribution is presented in Fig. 4 ▸(*f*) (continuous line). Surprisingly, the obtained scattered intensity, shown in Fig. 4 ▸(*e*) (black circles), was satisfactorily fitted using both models (red and blue continuous lines). Nevertheless, while the SBA-15+MC model can successfully retrieve the size distribution, as shown in Fig. 4 ▸(*f*), the SBA-15 model fails, as clearly shown by the fit parameter values (Table 3 ▸).

This result highlights two important points. The first is related to the fact that SAS is a low-resolution technique (Oliveira, 2011 ▸), meaning that different models might fit the available data quite well. To prevent, or at least to mitigate, ambiguity in the information obtained from different fit procedures, one needs additional information on the system under investigation, for example, the pore size distribution from NAI. This implies that the most appropriate model to analyse the data can be unambiguously selected, leading us to the second point, which demonstrates the importance of the constraint imposed by the analytical function 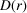 on the SBA-15 model and how it can propagate deviations in the remaining fit parameters, as one can observe from Table 3 ▸. In this specific example, besides the clear differences in *R* and 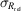 values, we noticed significant deviation in the values of the parameters *T*, 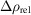 , 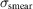 , 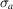 , 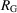 , Sc1, Sc2 and AP, most of them being overestimated in relation to the input values. This might affect the interpretation of the obtained structural data. Therefore, even if both models can satisfactorily fit the data, the misuse of the SBA-15 model is clear – it simply cannot represent the system. At this point, as mentioned in Section 1, one could modify the equations of the SBA-15 model, on the basis of prior information, to properly describe the new features of the system. Fortunately, this task is unnecessary if one uses the SBA-15+MC model proposed in this work.

## 5. Conclusion

In this work we proposed, for the first time, a flexible version of one of the state-of-the-art models used to analyse SAXS data of SBA-15, referred to here as the SBA-15 model; by using an MC method, it is now possible to recover the size distribution 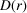 of mesopores without any prior information, *i.e.* without using an analytical expression. The new method, called the SBA-15+MC model, was validated using simulated data and used to successfully retrieve the mesopore size distribution along with other structural features from experimental data of SBA-15, demonstrating its applicability and robustness. To achieve these targets, we needed to adapt MC equations to work with long core–shell cylinders and optimize the processing of the SBA-15 model by simplification and the determination of structural information from the SAXS curve beforehand that may then be added to the fit procedure *a posteriori*. This improved the processing of the model in terms of speed and stability, since it also reduced the number of fit parameters, all constrained to ensure physically meaningful results. Equally important is the constraining of the radius sampling interval of the MC procedure, 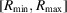 , to guarantee the convergence of the method. For conventional SBA-15, an interval between *R*_min_ = 20 Å and *R*_max_= 80 Å has proven to be satisfactory for a typical *q* range between 0.01 and 0.3 Å^−1^. With the new method, we have also shown that the SBA-15 model only works because the SBA-15 mesopore size distribution is narrow. The SBA-15+MC model was successfully applied to model SBA-15 with expanded mesopores, while the SBA-15 model, despite yielding satisfactory fits, failed to recover the correct size distribution and the values of the other fit parameters. In fact, the increase of mesopore width is followed by a decrease in the structural order (Garcia *et al.*, 2016 ▸), but this is necessary to incorporate molecules of larger sizes, for instance diphtheria and tetanus anatoxins (Trezena *et al.*, 2022 ▸).

Following the strategies provided in this work, as demonstrated by the tests performed using both simulated and experimental data, we have shown that we were able to successfully retrieve the mesopore size distribution of SBA-15 in a free modelling approach. Furthermore, since equation (32) is generally written for any 2D-hexagonal arrangement of long core–shell cylindrical mesopores, one can use the SBA-15+MC model to analyse, for instance, modified syntheses of SBA-15 (Losito *et al.*, 2021*a* ▸; Guo *et al.*, 2019 ▸) and similar OMMs such as MCM-41, SBA-3, FSM-16 and AMS-3 (Chew *et al.*, 2010 ▸). Adaptations of equation (32) to include different structure factors, keeping the MC part, are also possible. For instance, changing the lattice from 2D-hexagonal to 2D-square packing (*P*4*mm* space group) (Förster *et al.*, 2005 ▸), one could, in principle, fit SAXS data from DNA–silica complexes (Jin *et al.*, 2009 ▸). This demonstrates that, even though we chose a specific SAXS model to work with, the strategies presented herein are general, thus opening new opportunities for inclusion in other models aimed at the analysis of SBA-15 and other OMMs. In the future, similar strategies can also be easily applied for other systems, combining form factors and structure factors, which certainly opens a broad range of applications.

The program code as well as the measured and simulated data sets will be freely supplied by the authors upon request for inspection, improvements and application under a Creative Commons Attribution Share-alike licence. Because it is written in Python, the code can be easily combined with other routines, allowing its use in high-throughput analysis and machine learning methods, which is a very important demand on fourth-generation synchrotrons.

## Figures and Tables

**Table 1:** Parameters of the SBA-15 model and their description Entries marked with ‘*’ can be estimated in advance, *i.e.* prior to fitting of the data with the SBA-15 model, as discussed in the text.

Parameter	Description
*R*	Mesopore inner radius
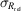	Relative polydispersity of the radius, 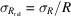
*T*	Mesopore shell thickness
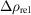	Electron-density contrast of the core relative to the shell [equation (7)]
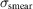	Smearing width of the mesopore shell
(*) 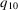	Position of the peak 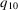 , related to the lattice parameter *a* by equation (19)
(*) 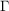	FWHM of the peak, the same for all peaks in the SAXS curve
(*) 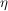	Fraction of the Lorentz function in the pseudo-Voigt function, varying from 0 to 1
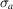	Quantifies the distortion relative to an ideal 2D-hexagonal lattice, being zero for an ideal lattice
(*) 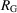	Radius of gyration related to the polymer-like scattering at high *q*
Sc1	Scale factor relative to the mesopore contribution to the total scattering
(*) Sc2	Scale factor relative to the micropore contribution to the total scattering
(*) Back	Constant incoherent scattering contribution
(*) AP	Scale factor relative to the Porod law contribution which considers the interface between the grains

**Table 2:** Code blocks of the general flowchart (Fig. 3 ▸) for processing of the cylinder+MC, cylinder-CS+MC and SBA-15+MC models

Code block	Cylinder+MC	Cylinder-CS+MC	SBA-15+MC
1	Compute 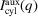 , equation (25)	Compute 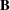 and 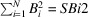	Compute 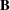 , 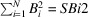 and 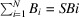
			
2	Fit data with 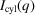 , equation (26) → [sc, back], 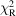	Fit data with 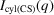 , equation (28), with 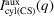 given by equation (31) → [sc, back, *T*, 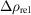 ], 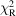	Fit data with 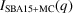 , equation (32), with 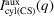 given by equation (31) → [Sc1, *T*, 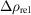 , 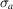 , 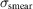 ], 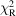
			
3	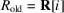	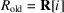	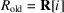
	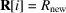	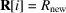	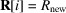
	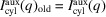	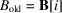	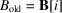
	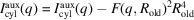 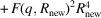	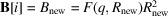	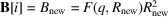
		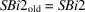	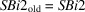
		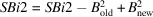	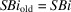
			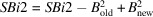
			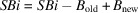
			
4	Fit data with 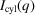 , equation (26) → [sc, back], 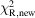	Fit data with 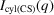 , equation (28), with 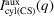 given by equation (31) → [sc, back, *T*, 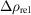 ], 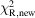	Fit data with 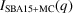 , equation (32), with 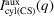 given by equation (31) → [Sc1, *T*, 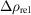 , 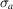 , 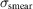 ], 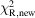
			
5	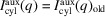	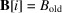	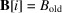
	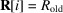	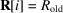	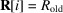
		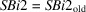	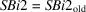
			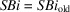

**Table 3:** Summary of the tests performed with the SBA-15+MC model using simulated and experimental data Uncertainties in the last digit, when present, are given in parentheses.

	Simulated data		Experimental data		Simulated data (expanded mesopores)		
Parameters	Input values	SBA-15+MC model ( 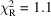 )	SBA-15 model ( 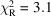 )	SBA-15+MC model ( 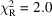 )	Input values	SBA-15 model ( 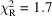 )	SBA-15+MC model ( 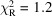 )
*R* (Å)	45.0	–	47 (1)	–	–	57.3 (7)	–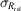 (%)	5.0	–	8(1)	–	–	23.0 (4)	–
*T* (Å)	30.0	29.69 (2)	28 (2)	27.3 (3)	30.0	20.6 (5)	27.3 (6)
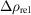	0.0	0.0011 (1)	0.00 (3)	0.00 (1)	0.0	0.08 (1)	0.006 (7)
σ_smear_ (Å)	5.0	4.98 (1)	5(2)	6.4 (3)	5.0	30 (1)	7.0 (5)
*q*_10_ (Å^−1^)	0.06	0.060 (1)	0.0613 (2)	0.0612 (4)	0.045	0.045 (1)	0.0447 (2)
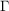	0.002	0.00195 (5)	0.0030 (4)	0.0031 (1)	0.004	0.0039 (2)	0.00385 (6)
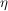	0.4	0.379 (5)	0.66 (2)	0.582 (1)	0.4	0.44 (1)	0.394 (2)
σ_*a*_ (Å)	0.05	0.0499 (5)	0.048 (3)	0.0451 (2)	0.07	0.040 (4)	0.068 (3)
*R*_G_ (Å)	8.0	8.2 (1)	8.4 (2)	9.35 (1)	8.0	10.2 (2)	7.96 (7)
Sc1	1.0 × 10^−3^	1.0 (3) × 10^−3^	689 (8)	690 (1)	1.0 × 10^−2^	155 (3) × 10^−2^	1.0 (2) × 10^−2^
Sc2	1.0 × 10^−5^	1.05 (5) × 10^−5^	6.5 (5)	8.76 (1)	1.5 × 10^−4^	400 (2) × 10^−4^	1.49 (4) × 10^−4^
Back	1.0 × 10^−7^	1.29 (4) × 10^−7^	0.00 (1)	0.006 (1)	1.0 × 10^−7^	0.0 (1) × 10^−7^	1.0 (1) × 10^−7^
AP	5.0 × 10^−11^	5.15 (3) × 10^−11^	1.09 (6) × 10^−6^	1.57 (2) × 10^−6^	5.0 × 10^−11^	759 (4) × 10^−11^	5.96 (3) × 10^−11^
